# Cutaneous wound healing: canine allogeneic ASC therapy

**DOI:** 10.1186/s13287-020-01778-5

**Published:** 2020-06-29

**Authors:** Nathaly Enciso, Luis Avedillo, María Luisa Fermín, Cristina Fragío, Concepción Tejero

**Affiliations:** 1 “Experimental Hematology” UCM-Research Group, Veterinary Faculty, Complutense University of Madrid, Avda Puerta de Hierro s/n, 28040 Madrid, Spain; 2 Department of Biochemistry and Molecular Biology, Veterinary Faculty, Complutense University of Madrid, Avda Puerta de Hierro s/n, 28040 Madrid, Spain; 3 Department of Animal Surgery and Medicine, Veterinary Faculty, Complutense University of Madrid, Avda Puerta de Hierro s/n, 28040 Madrid, Spain

**Keywords:** Adipose mesenchymal stem cells, Canine cutaneous wounds, Regenerative medicine, ASC therapy

## Abstract

**Background:**

Wound healing is a complex biological process comprised of a series of sequential events aiming to repair injured tissue. Adult mesenchymal stem cells (MSCs) have been used in cellular therapy in preclinical animal studies; a promising source of MSCs is adipose tissue (AT). In this paper, we evaluated the clinical value and safety of the application of cultured allogenic MSCs from AT for acute and chronic skin wound healing in a canine model.

**Methods:**

Twenty-four dogs of different breeds between 1 and 10 years of age with acute and chronic wounds were studied. Morphology of the wounded skin was monitored for changes over time via serial photographs and histopathological studies.

**Results:**

The percentage of the wounds that exhibited contraction and re-epithelialization were significantly different between wounds treated with adipose mesenchymal stem cells (ASCs) and control wounds; this effect was observed in both acute and chronic conditions. At 90 days, re-epithelization of acute and chronic wounds reached more than 97%. Histopathological study revealed a reduction in inflammatory infiltrate and the presence of multiple hair follicles on day 7 after treatment with ASCs, promoting epidermal and dermal regeneration. To guarantee the safety of our treatment, we determined the serum levels of cytokine markers in our patients. ASC treatment upregulated granulocyte-macrophage colony stimulating factor (GM-CSF) at the gene level, which may contribute to the recruitment of cells that participate in skin repair to the site of injury.

**Conclusions:**

The development of an allogenic ASC therapy to improve wound healing in a canine model could have a clinical impact in human treatment.

## Background

Adult mesenchymal stem cells (MSCs) have been widely used in regenerative medicine in both in vitro and in vivo preclinical research and clinical trials. MSCs have high proliferation potential, self-renewal abilities, and multilineage differentiation capabilities enabling them to produce cells of mesodermal and non-mesodermal origin; these cells are able to secrete soluble factors that stimulate the migration, mitosis, and differentiation of local stem cells. These characteristics make MSCs interesting tools for tissue engineering and regeneration in human clinical trials.

In recent years, an increasing number of studies have arisen, with different approaches like comparing the efficacy of allogeneic and autologous MSCs, the origin of the sample, the method of MSC isolation [1–4], and the determination of the dose and adjuvant products [5, 6], because these therapies are not yet fully standardized. In the European Union, the use of these treatments has been possible because cell therapy products have been considered medicinal products since 2003, according to legislation through Directive 2003/63/EC [7].

A major advance has been the development of MSC therapies to treat a variety of human diseases [8, 9]. The protocols have been developed due to the fact that MSCs possess unique characteristics, such as hypoimmunogenicity, immunomodulation, and anti-inflammatory properties, which make them interesting and safe tools to be explored for possible therapeutic uses.

A number of studies confirmed the existence of MSCs in canine adipose tissue as well as in bone marrow [10–13]. In preclinical animal studies, a promising source of MSCs has been found to be adipose tissue (AT). In fact, adipose mesenchymal stem cells (ASCs) are being used, especially in small breeds where the amount of bone marrow that can be harvested is limited and the collection is more laborious and painful.

In view of future therapeutic applications, the study of the expression of specific differentiation-related genes is a pivotal prerequisite. Recent studies have explored the role of transcription factors, including *Runx2*, *Sox9*, and *PPARγ*, in the differentiation of MSCs [14–17]. The overexpression of a single transcription factor in MSCs may promote transdifferentiation into a specific cell lineage, which could then be used for the treatment of some diseases. In this context, it is crucial to use stable housekeeping genes (HGs) for normalization of RT-qPCR data to obtain validated and comparable results [18, 19]. Wound healing is a complex biological process comprised of a series of sequential events aiming to repair injured tissue [20–22]. During these processes, growth factors, cytokines, matrix metalloproteases, and angiogenesis factors play an important role in processes like inflammation, formation of granulation tissue, re-epithelialization, matrix formation, and re-modeling [23].

The cellular and molecular mechanisms supporting tissue repair are still poorly understood, and current therapies are limited [24–27]. Previous studies of MSC transplantation in animal models and human patients have demonstrated improved therapeutic effects in terms of rapid wound healing and improved dermal regeneration [7, 28–33]. After systemic administration, MSCs are also capable of migrating to and engrafting in sites of inflammation where they exert local functional effects in the resident tissue [34–38]. In addition, MSCs regulate immune and inflammatory responses and can have a reparative effect through paracrine signaling by releasing biologically active molecules that affect cell migration, proliferation, gene expression, and survival of the surrounding cells [33, 39]. Studies have demonstrated that treatment with MSCs has significant immunomodulatory effects during wound healing [40, 41]. This immunomodulatory consequence on the host makes them a suitable candidate for allogeneic transplantation. Allogenic MSC administration has the advantage of prompt preparation of material, and it does not depend on the health status of the patient [42–44].

In this context, the dog is considered an ideal research model because it is a large animal that shares the same environment as humans and develops many diseases that occur in humans, and dog’s immune system is known to be similar to those of humans [45, 46]. In addition, wound healing has been studied using dogs as a translational model for both veterinary and human applications [47].

Identification of the positive and adverse effects of allogenic MSC transplantation for skin wound healing using a canine model is needed prior to its use in human clinical trials.

In this paper, we evaluated the clinical value and safety of the application of cultured adipose allogenic mesenchymal stem cells for treating acute and chronic skin wound healing in a canine model. We estimated serum levels of cytokine markers to ensure the welfare and security of our treatment. Wound skin morphology and changes over time were monitored via serial photographs and histopathological studies, and wound closure areas were estimated. In addition, key transcription factors involved in the differentiation of mesenchymal stem cells were evaluated.

We hypothesized that ASC-treated wounds could have improved outcomes compared to untreated controls through local mRNA expression of several factors related to cutaneous wound healing.

The research presented in this study can direct us toward improved therapeutic protocols.

## Methods

Twenty-four dogs of different breeds that were between 1 and 10 years of age that presented with acute and chronic wounds produced by sports activities as well as household injuries were included in this study, wounds penetrating into the subcutaneous tissue without muscular tissue affection. Wounds produced by bites were excluded. Eight healthy dog skin biopsies were taken for RT-qPCR analysis. All animal experiments should comply with the Animal Research: Reporting In Vivo Experiments (ARRIVE) guidelines and were approved by the ethical committee of our institution (approval no. 08/2017). Dog owners provided informed consent for the treatment. The study was performed on 24 dogs divided into four groups: group I, 6 dogs with acute skin wounds treated with conventional treatment; group II, 10 dogs with acute skin wounds treated with ASCs; group III, 4 dogs that had chronic skin wounds for 1–2 months treated with conventional treatment; and group IV, 4 dogs that had chronic skin wounds for 2–3 months that were then treated with ASCs.

### Treatment protocols

All dogs were treated every 48 h for 8 days with 15 mg/kg IM amoxicillin trihydrate (Bivamox® L.A.; Boehringer Ingelheim España, S.A.) and 5 mg/kg every 24 h PO enrofloxacin (Ganadexil enroflocaxino; Industrial Veterinaria, S.A., Barcelona, Spain). After 3 days with this treatment protocol, one dose of 3 × 10^7^ allogeneic ASCs in phosphate-buffered saline (PBS; as a vehicle) was injected intradermally around wounds with an area of up to 10 cm^2^, and two doses of 3 × 10^7^ cells were injected in wounds with an area that was greater than 10 cm^2^. The first injection took place at day 3 and the second dose at day 10 after wounding. Groups I and III received conventional treatment with an ointment containing *Centella asiatica* extract and neomycin (Blastoestimulina® 1%; Almiral S.A., Barcelona, Spain) until the wound was healed.

### Isolation, expansion, and differentiation of adipose MSCs

ASCs were obtained as previously described by Enciso et al. [10]. Briefly, omental adipose tissue was obtained from healthy donor female dogs that were undergoing elective sterilization, and ASCs were consecutively isolated and cultured in DMEM that contained 10% canine serum (dog serum was derived from pooled batches obtained from whole blood collected in tubes free of anticoagulant, centrifuged at 2000 rpm for 10 min, and filtered through 0.2 μm) and 1% antibiotic; the supernatant obtained after 3 weeks from the culture was considered conditioned medium (CM) and was frozen at − 80 °C. ASCs used for injection were cultured in the Luria-Bertani medium (Scharlab S.L. Barcelona, Spain) to evaluate sterility. Before using the ASCs therapeutically, cells were analyzed by flow cytometry to confirm that they were positive for CD90 and negative for CD34, CD45, and MCH-II as previously reported [10, 48]. Their ability to adhere to plastic and their fibroblast-like morphology were confirmed. These criteria of MSC were defined by the International Society for Cellular Therapy [49].

### Differentiation

The multipotentiality of ASCs was determined by analyzing their capacity to differentiate into three lineages.

#### Osteogenic differentiation

Osteogenesis differentiation medium (StemPro, Gibco) was used according to the manufacturer’s instructions. ASCs were cultured for 21 days, the medium was changed every 3rd day, and differentiation was assessed by von Kossa staining. For this process, the cells were fixed with 4% formaldehyde solution for 30 min, which was followed by rinsing with PBS and incubation with 2.0% silver nitrate in the dark for 30 min. After rinsing with distilled water and air drying, the cells were exposed to UV light for 60 min to develop calcium phosphate precipitate. The cells were washed several times with PBS and were visualized under a light microscope. Brown staining indicated the deposition of calcium phosphate precipitate by osteoblasts.

#### Chondrogenic differentiation

Chondrogenesis differentiation medium (StemPro, Gibco) was used according to the manufacturer’s instructions. ASCs were cultured for 14 days, the medium was changed every 3rd day, and the differentiation was assessed by Alcian blue staining. The cells were fixed with 4% formaldehyde for 30 min and then were washed with PBS. Then, 1% Alcian blue, which was prepared in 0.1 N HCl, was added for 30 min incubation. Finally, the ASCs were washed with 0.1 N HCl pH 1.0, and distilled water was added. Blue staining indicated chondrocyte synthesis of proteoglycans.

#### Adipogenic differentiation

An adipogenesis differentiation kit (StemPro, Gibco) was used according to the manufacturer’s instructions. ASCs were cultured for 21 days, the medium was changed every 3rd day, and differentiation was assessed by the presence of lipid droplets that were visualized after staining with oil red O solution. The cells were fixed with 10% formal calcium fixative for 60 min, and then, they were washed with PBS and then with 70% ethanol. Addition of oil red O solution was followed by rinsing the cells with 70% ethanol and then with tap water. Red staining indicated the presence of lipids.

### Transwell assay

Migration assays were conducted using 8 μM Transwell plates (Corning). ASCs at passage 3 were seeded in triplicate at a density of 3 × 10^3^ cells/Transwell in DMEM in the upper chamber of Transwell plates. We evaluated the following solutions: DMEM, DMEM + 10% canine serum, DMEM + granulocyte-macrophage colony stimulating factor (GM-CSF) (100 or 200 μg/ml), and conditioned medium (CM). The cells were placed in the lower chamber to induce cell migration. After 8 h, cells on the top of the Transwell were removed, and cells that had migrated to the lower surface were fixed by incubation with cold 70% ethanol (4 °C) for 10 min (room temperature), and then, they were incubated with the May-Grünwald-Giemsa staining solution. Images of cells were captured using an inverted microscope (Leica) and were subsequently manually quantified.

### Multiplex assays

Peripheral blood from each dog was collected in sterile tubes for cytokine analysis. Serum was separated by centrifugation and then was stored at − 80 °C. Serum samples were batch analyzed at the conclusion of the study. Bead-based multiplex assays measure multiple cytokines from the same sample at the same time. Analyses were performed according to the manufacturer’s instructions, with internal quality control using a Milliplex MAP Canine kit with Luminex technology (Immunology Multiplex Assay). The following cytokines were measured: granulocyte-macrophage colony stimulating factor (GM-CSF), interleukin-6 (IL-6), interleukin-7 (IL-7), interleukin-8 (IL-8), interleukin-10 (IL-10), interleukin-15 (IL-15), interleukin-18 (IL-18), interferon ɣ-inducible protein-10 (IP-10), keratinocyte chemoattractant (KC), monocyte chemoattractant protein-1 (MCP-1), and tumor necrosis factor α (TNFα).

### Clinical evaluation

Lesion progression was documented using a ruler to measure wound size. Photographs were taken at 7, 30, and 90 days post-treatment. The percent wound size and epithelization were evaluated according to the criteria proposed by Farghali et al. [50].

### Skin biopsies for histological analysis and gene expression

On day 7 after initiation of treatment, 4-mm-diameter punch biopsies of the ASC-treated wound, conventionally treated wound, and normal skin were taken from the edge of wounds for histopathological study and RT-qPCR analysis. Formalin-fixed and paraffin-embedded skin biopsy samples were stained with hematoxylin and eosin (H&E) and were examined under a light microscope (Leitz Laborlux S).

### RT-qPCR analysis

Total RNA was extracted from 10^6^ cultured ASCs using an RNeasy® Mini kit (Qiagen, Hilden, Germany) according to the manufacturer’s protocol. Total RNA from tissue was extracted using TRIzol (Invitrogen); briefly, 50 mg of tissue was homogenized and incubated for 5 min at 30 °C, and then, chloroform was added and incubated with the homogenized tissue at 30 °C for 3 min. The sample was centrifuged for 15 min at 12,000×*g* at 4 °C, and the aqueous phase containing the RNA was transferred to a new tube and treated with RNeasy Mini kit (Qiagen) according to the manufacturer’s protocol. Reverse transcription was performed with 1 μg of total RNA and a “High Capacity RNA-to-cDNA Kit” (Applied Biosystems, Foster City, CA, USA) according to the manufacturer’s protocols. RT-qPCR was performed with a “Power SYBR® Green PCR Master Mix” (Applied Biosystems) on QuantStudio 12k Flex equipment (Applied Biosystems). All nucleotides were purchased from Metabion International AG (Munich, Baviera, Germany). A set of primers was designed to amplify the following canine genes: *TBP*, *RUNX2*, *SOX9*, *PPARG*, *MMP2*, *GM-CSF*, *VEGFA*, and *IL-10*.

Primer designs were based on the Primer3 program [51] and the National Center for Biotechnology Information Blast Search Program (http://www.ncbi.nlm.nih.gov/).

The primers were as follows:

**Table:** 

Genes	Primer sequences
*TBP*	Forward: 5′-CCGTCTATCTGAACTGGGAAA-3′
	Reverse: 5′-AAGGGTCATGAGTGGCATGT-3′
*RUNX2*	Forward: 5′-TGAGCACCGAAGAACAACTG-3′
	Reverse: 5′-GCTGCTGCTGCTACACTGAC-3′
*SOX9*	Forward: 5′-AGCGAACGCACATCAAGAC-3′
	Reverse: 5′-GAGGCTGAAGGGGCTGTAG-3′
*PPARγ*	Forward: 5′-TGGCAAAGAGCTGAGAGGAC-3′
	Reverse: 5′-AAAATCAAGTTCAAACACATCACC-3′
*MMP2*	Forward: 5′-GAGCGAGGGTACCCCAAG-3′
	Reverse: 5′-GCTCCAATTAAAGGCAGCAT-3′
*GM-CSF*	Forward: 5′-TCTCTGAAGTGTTTGACCCTGA-3′
	Reverse: 5′-CAGGCCCTCCTTGTACAGC-3′
*VEGFA*	Forward: 5′-CGTGCCCACTGAGGAGTT-3′
	Reverse: 5′-GCCTTGATGAGGTTTGATCC-3′
*IL-10*	Forward: 5′-CAGGTGAAGAGCGCATTTAGT-3′
	Reverse: 5′-TCAAACTCACTCATGGCTTTGT-3′

The PCR protocol started with one cycle at 95 °C for 10 min and continued with 40 cycles of 95 °C for 15 s and 60 °C for 1 min. Assays were performed in duplicate, and the average threshold cycle number (Ct) for each tested gene and condition was used to quantify the relative gene expression according to the ΔΔCt method. Normalization was performed using *TBP* as a housekeeping gene [18, 52]. To confirm the specificity of the primers used, the obtained amplicons were compared with the target gene sequences available in the GenBank/EMBL databases using Blast software (http://www.ncbi.nlm.nih.gov/BLAST).

### Statistic

The results were analyzed using the software programs SPSS 25 (IBM Corporation, Endicott, NY, USA) and Graph Pad Prism version 6. Normally distributed data were assessed using the Kolmogorov-Smirnov and Shapiro-Wilk tests. Gene expression along differentiation and gene expression in skin wound by RT-qPCR were evaluated by ANOVA post-Bonferroni test. Migration was evaluated by comparing the number of ASCs between groups with ANOVA post-Dunnett test. Serum cytokine levels were evaluated with ANOVA post-Tukey’s multiple comparisons test between dogs undergoing conventional treatment or ASC treatment at 7 and 30 days post-treatment. The percentage of regenerated area of acute and chronic wounds after conventional and ASC treatment was calculated with ANOVA post-Bonferroni test. All are expressed as the mean ± SD. Differences were considered significant when *p* < 0.05.

## Results

### Differentiation and transmigratory capacity of ASC

**Fig. 1:**
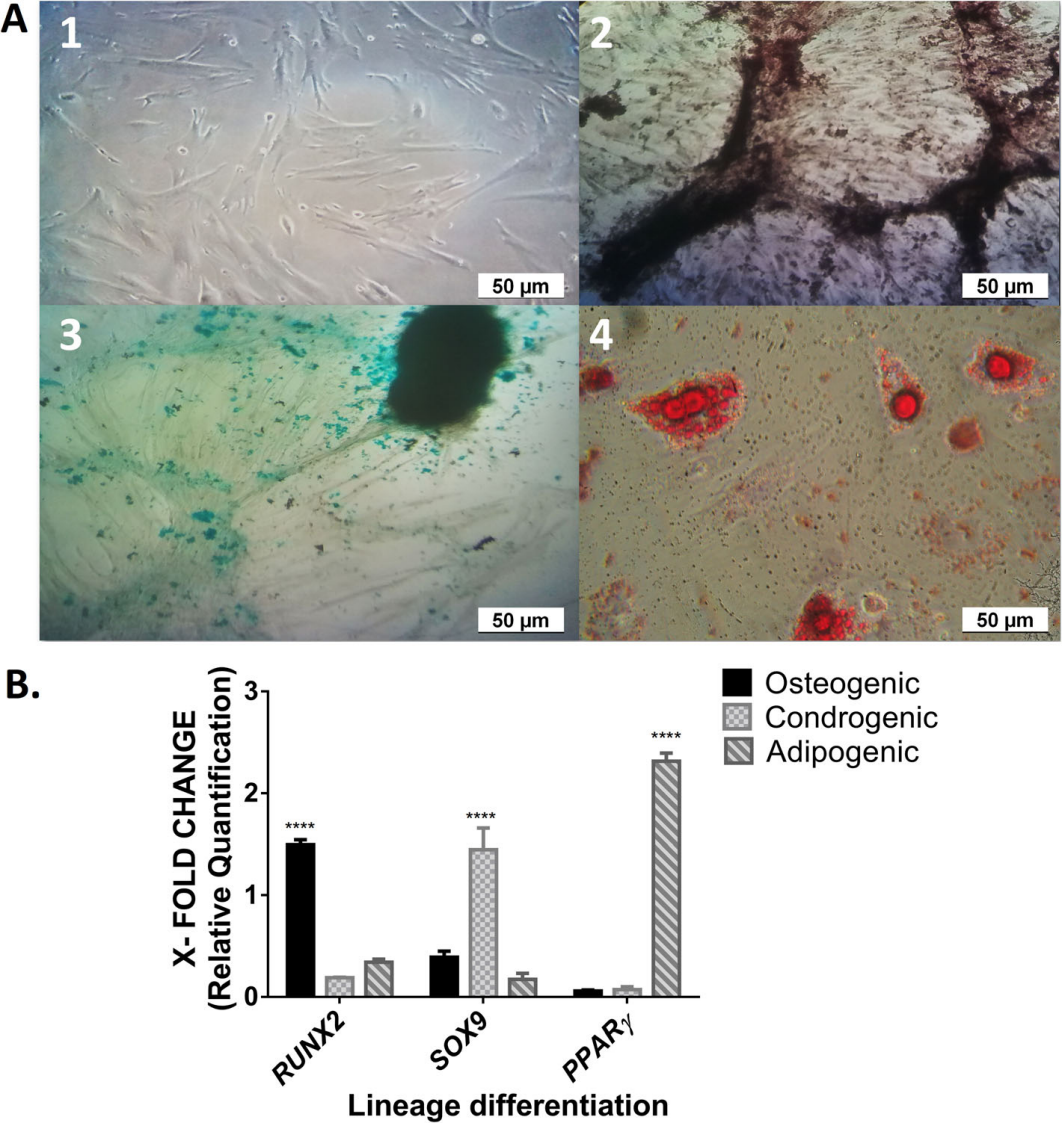
Differentiation of canine adipose-derived mesenchymal stem cells (ASCs) into osteocyte, chondrocyte, and adipocyte lineages. **a** Representative photographs. ASCs from the 3rd passage showed spindle-shaped fibroblastic cell morphology (1). After 3 weeks, ASCs differentiated according to the conditioned medium added. Cells underwent von Kossa staining for osteogenic lineage (2), Alcian blue staining for chondrogenic lineage (3), and oil red O staining for adipogenic lineage (4) (bar length = 50 μm). **b** RT-qPCR analysis of transcription factors in the differentiation of ASCs. Data represent mean fold change values in genes ± SD compared to ASCs. ****A statistically significant difference of *p* < 0.0001 of each transcription factors representative of a specific lineage with respect to the genes of other lineages. *n* = 3 different experiments

Trilineage differentiation capacity of ASCs is shown in Fig. 1a. When evaluating the transcription factors, we observed that *PPARɣ* showed a 2-fold increase in adipogenic differentiation compared to ASC, while *SOX9* and *RUNX2* did not show this augment in their representative linages; however, their increases were significant (*p* < 0.0001) with respect to other lineages (Fig. 1b) [14]. The values were normalized to the *TBP* gene and were compared to ASCs.

**Fig. 2:**
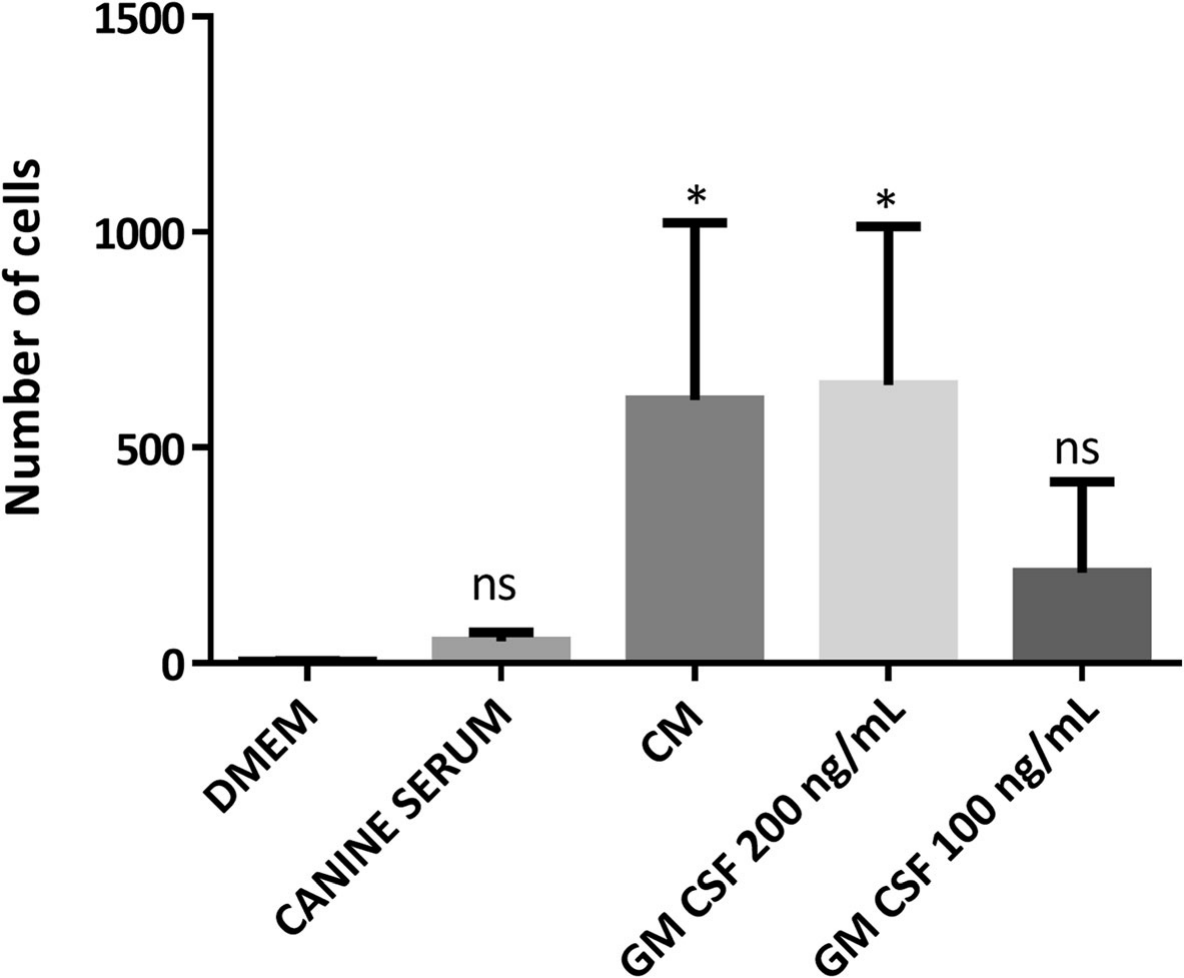
Transmigration capacity. Quantification of cells migrating toward DMEM, DMEM + canine serum 10%, conditioned medium (CM), DMEM + GM CSF 200 ng/ml, and DMEM + GM CSF 100 ng/ml. Data represent the mean ± SD. *p < 0.05. ns, not significant. *n* = 3 different experiments

The effect of GM-CSF on hematopoietic stem cell migration is well known, so we evaluated this effect on ASCs. At a dose of 200 ng/ml, there was an increase in the number of migrating cells compared to the 100 ng/ml treatment group. Interestingly, conditioned medium induced ASC migration effects that were similar to those following treatment with GM-CSF (200 ng/ml). Furthermore, both GM-CSF 200 ng/ml and conditioned medium produced significantly different (*p* < 0.05) migration results from those observed in the cells grown in DMEM. Our data represent the mean ± SD (Fig. 2).

### Evaluation of treatment safety by quantifying cytokine levels in serum

**Fig. 3:**
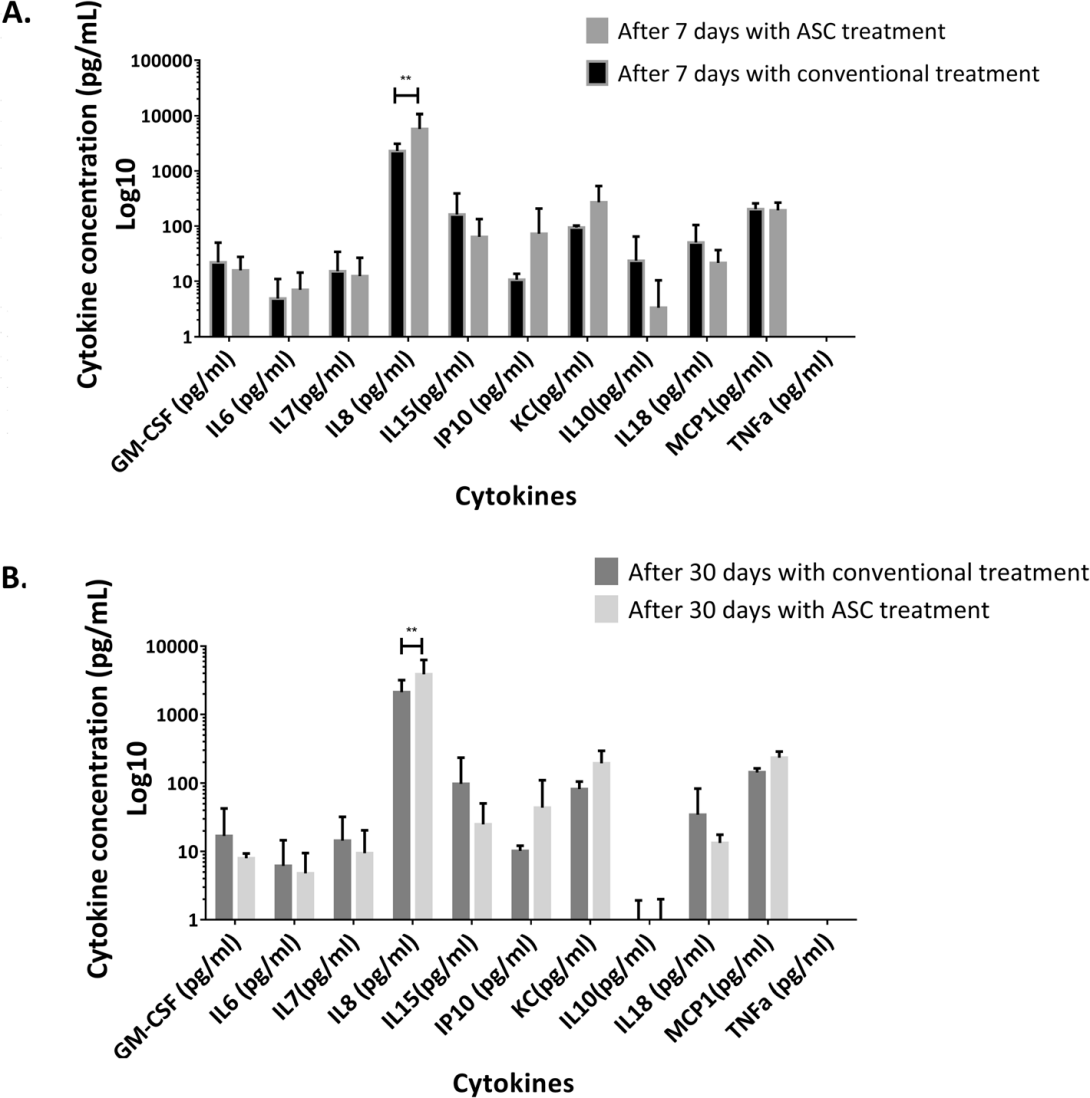
Cytokine concentration values in dogs using a Milliplex Canine Cytokine Panel. Canine serum levels of conventional and adipose-derived mesenchymal stem cell (ASC) treatment at 7 days (**a**) and 30 days (**b**) post-treatment. Values are the mean ± SD. **A statistically significant difference of *p* < 0.01. *n* = 4–6 for each experimental point

To estimate potential adverse events associated with the use of ASC treatment, we evaluated incidents according to the grading system of the veterinary cooperative oncology group [53]. We measured the cytokine profiles at 7 and 30 days post-treatment in sera from six patients treated with ASCs and four patients that underwent conventional treatment, and none of them did present adverse events during the study. Cytokine values in the serum were similar in patients with conventional and ASC treatment, except for IL8, which had a higher value in patients treated with ASCs than it did in the conventional treatment and was also significantly different (*p* < 0.01) at 7 and 30 days (Fig. 3); however, the IL8 mean value obtained at day 30 (3329 pg/ml) was within the normal range for healthy dog serum quantified by O’Neill et al., Kjelgaard-Hansen et al., and Safra et al. [54–56].

### Skin regeneration of cutaneous wounds by ASC therapy

**Fig. 4:**
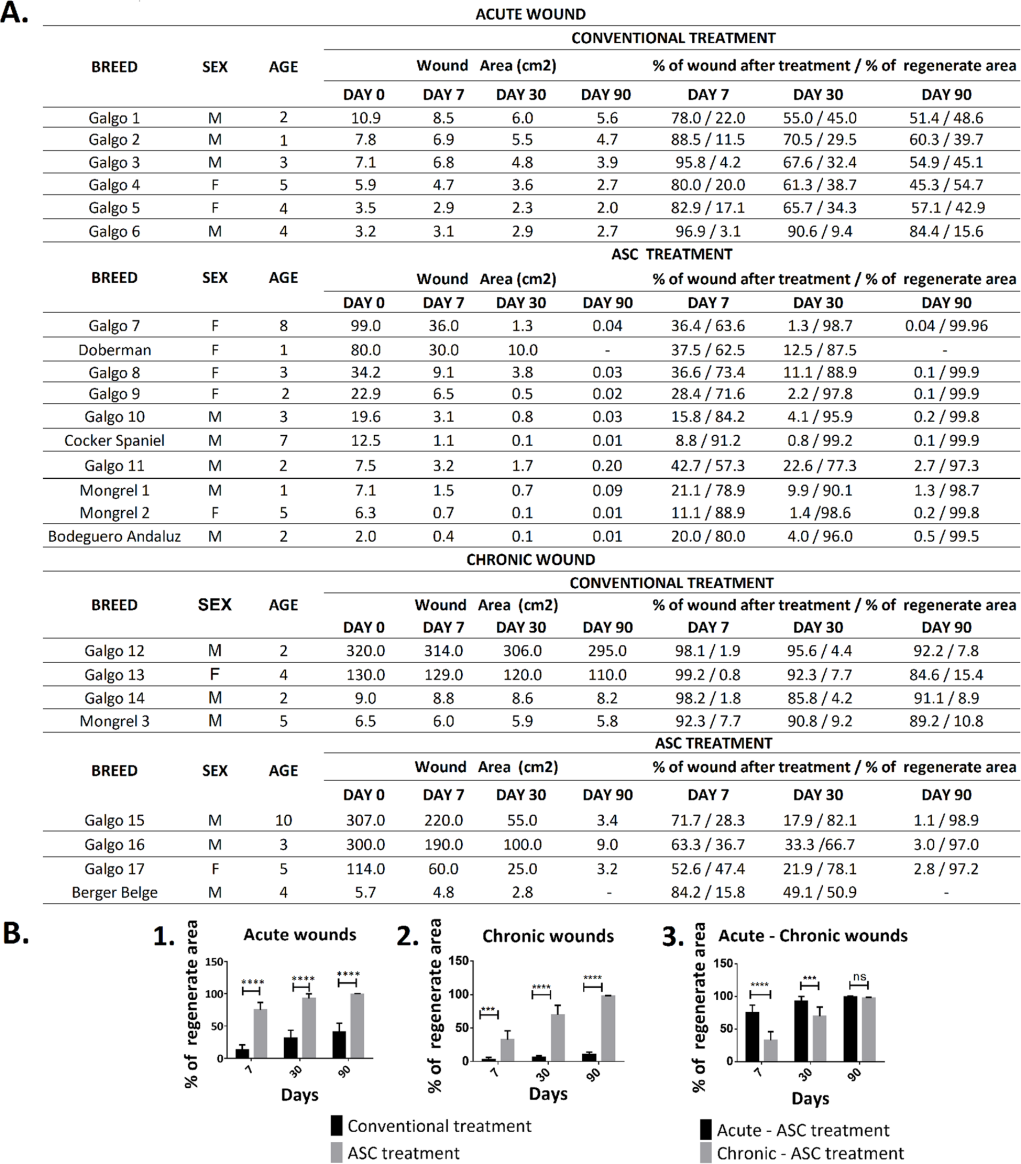
**a** Clinical evaluation. Wound contraction and regenerative area expressed in percentages in acute and chronic wounds. **b** Percentage of regenerated area of acute and chronic wounds after conventional and adipose-derived mesenchymal stem cell (ASC) treatment. Regenerated area for acute (1) and chronic (2) wounds treated with ASCs compared with conventional treatment at 7, 30, and 90 days. (3) Comparative studies between acute and chronic wounds treated with ASCs. Data values are the mean ± SD. ***A significant difference of *p* < 0.001. ****A significant difference of *p* < 0.0001. ns, no significant difference

The areas of the wounds were measured on the first, seventh, thirtieth, and ninetieth days after treatment. Wound contraction and regenerative area were calculated and expressed as percentages (Fig. 4a). The regenerated area in patients with acute wounds treated with ASCs at 7, 30, and 90 days was significantly different (*p* < 0.0001) from that of the time-matched conventional treatment (Fig. 4b (1)). Patients with chronic wounds treated with ASCs were significantly different at 7 (*p* < 0.001), 30, and 90 days (*p* < 0.0001) from those treated with the conventional treatment (Fig. 4b (2)). On the other hand, the percentages of regenerated area between acute and chronic wounds in patients treated with ASCs were significantly different at 7 (*p* < 0.0001) and 30 (*p* < 0.001) days, while on the ninetieth day, the regenerated area was similar (Fig. 4b (3)). In addition, we have analyzed data of acute wounds that have received one or two doses, obtaining the same significant difference *p* < 0.0001 (data not shown).

**Fig. 5:**
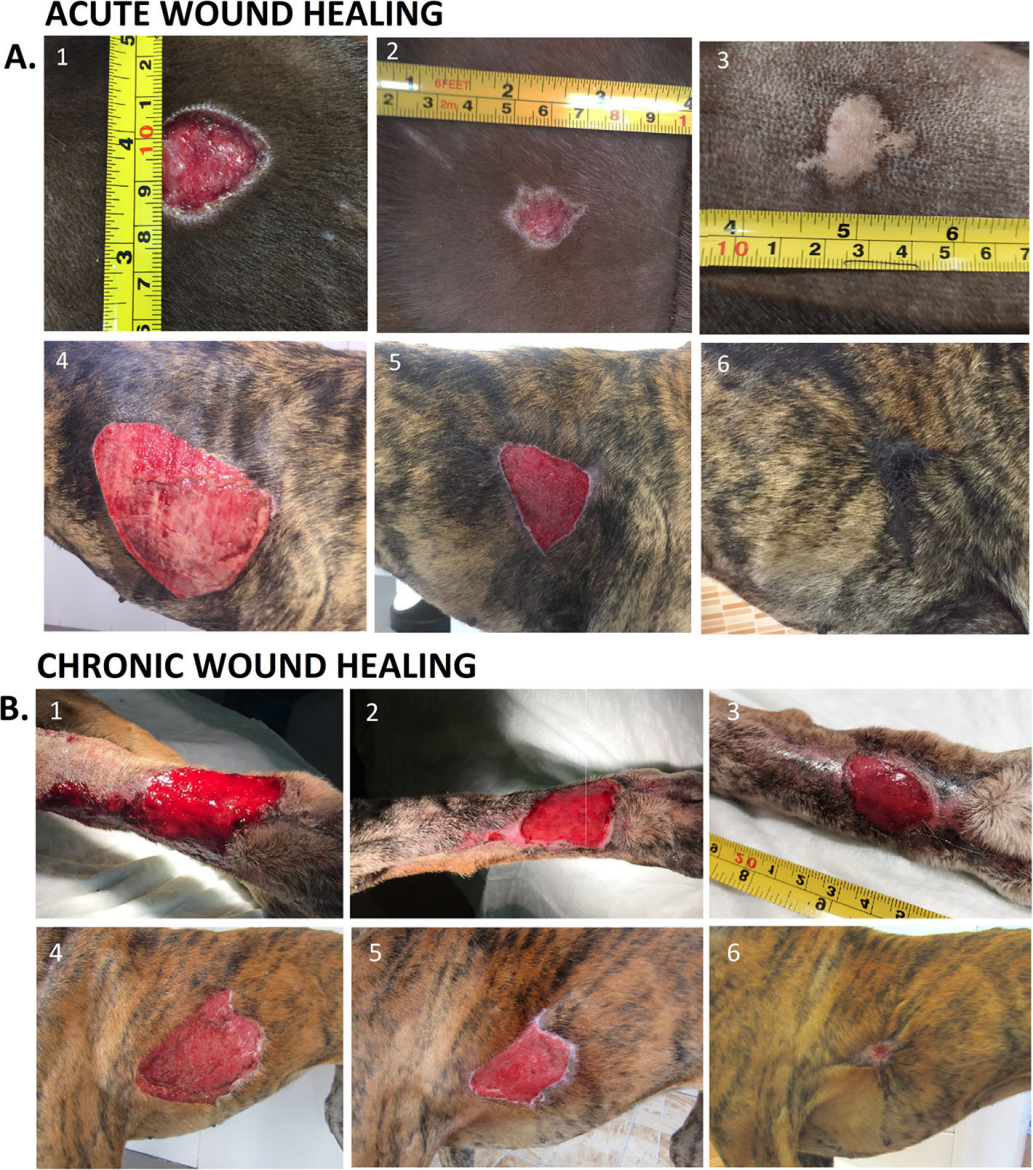
Re-epithelization of acute (**a**) and chronic (**b**) wounds is shown by representative photographs. Dogs with acute and chronic wounds treated with the conventional treatment (**a** (1–3), **b** (1–3)), and dogs treated with adipose-derived mesenchymal stem cells (ASCs) (**a** (4–6), **b** (4–6)) at day 0, 7, and 90

Re-epithelization was illustrated by photographs taken on the zero (Fig. 5a (1 and 4), b (1 and 4)), seventh (Fig. 5a (2 and 5), b (2 and 5)), and ninetieth (Fig. 5a (3 and 6), b (3 and 6)) days post-treatment of acute and chronic wounds, respectively. On day 7, acute and chronic wounds treated with ASCs (Fig. 5a (5), b (5)) displayed accelerated skin closure compared with conventional wounds (Fig. 5a (2), b (2)). On day 90, wounds with ASC treatment presented hair-bearing skin (Fig. 5a (6), b (6)); acute wound with conventional treatment had a common scar (Fig. 5a (3)); however, chronic wound presented unhealing wound (Fig. 5b (3)).

Histopathological evaluations were performed on the acute wound healing of eight Galgo Español dogs, four of whom were treated with conventional treatment and four with ASCs.

**Fig. 6:**
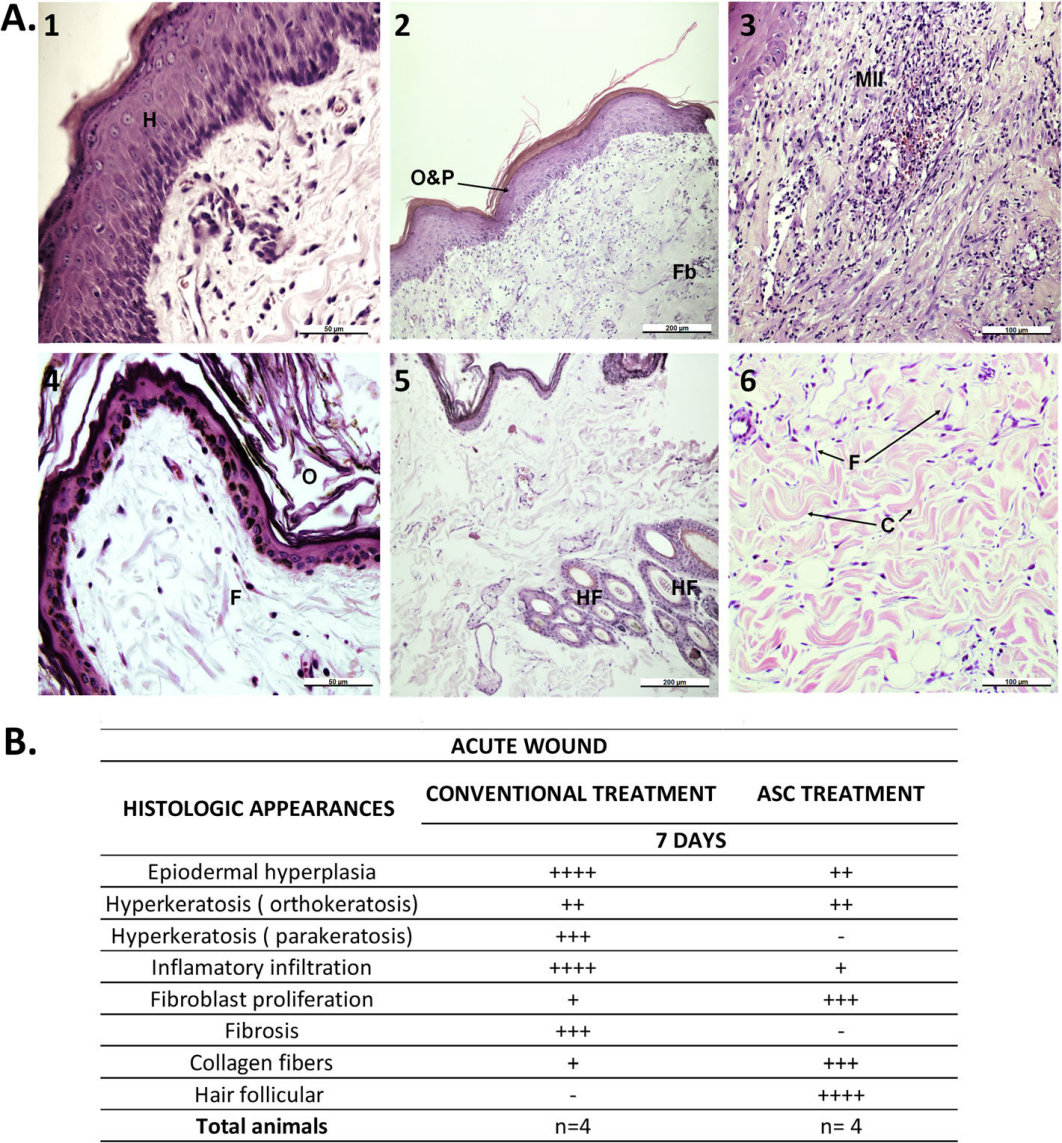
Histopathology of acute wound healing in control and ASC-treated skin wounds at 7 days post-treatment. **a** Representative photomicrographs demonstrate the histological characteristics of acute healing. H&E staining (bar length = 50 μm) (1 and 4), 200 μm (2 and 5), and 100 μm (3 and 6). (1–3) Control; (4–6) ASC-treated. H, hyperplasia; O, orthokeratosis; P, parakeratosis; Fb, fibrosis; MII, mononuclear inflammatory infiltrate; F, fibroblast; HF, hair follicles; C, collagen fibers. **b** Criteria for histological appearances in acute wounds with conventional treatment and ASC treatment. “-” and “+” = mild, “++” and “+++” = moderate, and “++++” = marked

The epidermis of the wound that received conventional treatment (Fig. 6a (1–3)) exhibited epidermal hyperplasia, hypergranulosis, hyperkeratosis, and ortho- and parakeratosis. The dermis exhibited fibrosis and an abundant mononuclear inflammatory infiltrate extending mainly through the dermis. These findings were consistent with acute dermatitis without follicular components, which is typical of a normal wound healing process. The wounds treated with ASCs (Fig. 6a (4–6)) exhibited relatively normal skin, thin epidermis, orthokeratosis, and fibroblast proliferation. In addition, multiple hair follicles in different stages of activity, collagen fibers, and a slight presence of inflammatory cells were observed in the dermis. The histological characteristics were classified according to the degree of severity: mild, moderate, or marked (Fig. 6b).

### Gene expression in the dynamic process of wound healing

**Fig. 7:**
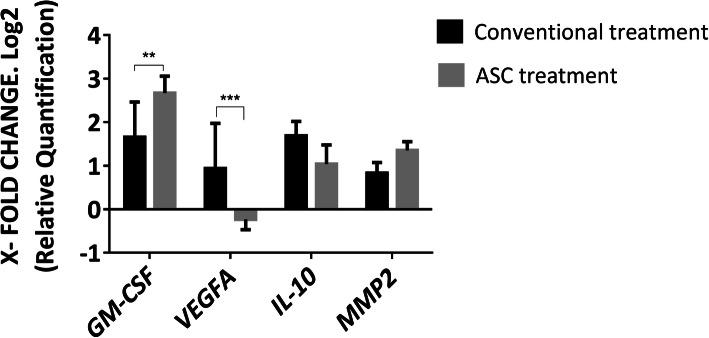
Relative quantification of gene expression in acute skin wounds after 7 days of conventional and ASC treatments. Data represent mean fold change values in genes ± SD for both treatments as compared to normal skin. Data values are the mean ± SD. **A significant difference of *p* < 0.01. ***A significant difference of *p* < 0.001

*GM-CSF*, *VEGF-A*, *MMP-2*, and *IL-10* genes were evaluated in sixteen patients. Ten dogs with acute wounds were treated with ACS, and six dogs with acute wounds were treated with conventional treatment. To compare our results with normal skin, eight biopsies were taken from healthy dogs. *GM-CSF* gene expression in dogs treated with ASCs shows an increase of more than 2-fold over wounds after that of conventional treatment with a *p* value < 0.01. *VEGFA* gene expression shows a decrease in ASC-treated dogs with a *p* value < 0.001. The expression of the *IL-10* and *MMP2* genes was similar in both treatments. The values were normalized to the *TBP* gene and were compared to normal skin (Fig. 7).

## Discussion

Wound healing requires a synchronized interplay among cells, growth factors, and extracellular matrix proteins. Several studies have demonstrated that mesenchymal stem cells coordinate the repair response by recruiting other host cells and secreting growth factors and matrix proteins.

Our study was performed to evaluate the role of allogeneic ASCs in acute and chronic wound healing therapies.

Considering this aim, there are essential characteristics that must be met to allow these cells to be candidates for cell-based therapy.

As we have previously mentioned, these cells can differentiate into multiple tissue forming cell lineages. During differentiation, upregulation or suppression of transcription factors occurs via specific signaling pathways. In osteogenic differentiation, *Runx2* is a key transcription factor that elevates osteoblast differentiation. Chondrogenic differentiation is driven by *Sox9*, and adipogenic differentiation is mainly controlled by *PPARγ*. We reported the expression of these transcription factor markers involved in the early differentiation of osteoblasts, adipocytes, and chondrocytes, and we presented images of differentiated cells (Fig. 1). These data are in agreement with the study performed by Almalki et al. [14].

As discussed in the “Methods” section, we utilized *TBP* gene for normalization of qRT-PCR data, according to Rangi et al. [18], who found that *TBP* has the top position for both geNorm and NormFinder analysis in MSCs, and Vandesompele et al., who found that TBP has the top position for geNorm analysis in skin wound [19].

Particular attention should be given to the homing capability of ASC, allowing the cells to find the way of injury and inflammation. Figure 2 shows the capacity of these cells under normoxic conditions when stimulated by conditioned medium as well as by the inflammatory cytokine GM-CSF to migrate and mediate regenerative effects at sites of tissue damage. The role of conditioned medium in increasing the transmigratory effect of ASCs is noted, which implies that in the process of proliferation, ASCs release factors and cytokines with chemoattractant activity. Enciso et al. [10] demonstrated the expression of MMP-2 and MMP-9 in ASCs enables the breakdown of the endothelial basement membrane.

Our pilot approach, reported by Enciso et al. [57], demonstrated a higher regenerative capacity with earlier and faster closure in wounds treated with ASCs in comparison to other forms of treatment.

In this paper, we extend this previous study to 24 dogs and evaluated the clinical value and safety of the application of cultured adipose allogenic ASCs for treating acute and chronic skin wound healing in the canine model described above.

Damage of the skin certainly induces local inflammation; this process involves multiple mediators, including chemokines, pro- and anti-inflammatory cytokines, and growth factors. To analytically evaluate the etiological role of inflammatory processes in systemic compartments, it is necessary to quantify the concentrations of relevant biomarkers in fluids, such as serum.

As shown in Fig. 3, of the cytokines studied, we did not observe increased levels in the serum of dogs treated with ASCs for 7 or 30 days. Although we observed a significant increase in IL-8 cytokine levels in wounds treated with ASCs at both 7 and 30 days, the levels of IL-8 (3858.64 pg/ml) in the serum of animals at day 30 post-treatment were similar to the median value (3329 pg/ml) of healthy dogs, which is data obtained with the multiplex assay by Safra et al., O’Neill et al., and Kjegaard-Hansen et al. [54, 55]. These results ensure the safety of our protocol, including potential effects on the immune system. This is of great importance when investigating new therapies, especially considering that many descriptive parameters of the dog’s immune system are quite similar to those of humans [45].

With respect to the histopathological study, Fig. 5a, b data support the active regenerative process, which revealed better organization of re-epithelialization, reduced inflammatory infiltrate, marked collagen fibers, and the presence of multiple hair follicles in different stages of activity on day 7 after treatment with ASCs by promoting epidermal and dermal regeneration.

These data indicate the success of allogeneic ASCs in wound healing therapy, providing faster wound healing and re-epithelization than that of other treatments.

Ribeiro et al. showed that the use of allogeneic MSC in chronic wounds in dogs was effective; however, unlike our study, at the end of the evaluation, they had the presence of scarring [58]. This may have been due to the fact that they used a low dose of stem cells (1 × 10^5^/cm^2^) compared to our study where we used 3 × 10^6^/cm^2^ and obtained a regeneration of the skin with characteristics similar to normal skin as well as reduction of scar formation.

Many clinical trials in wounds showed that the use of ASC therapy alone or combined with other compounds, such as platelet-rich plasma (PRP), hyaluronic acid, and others, can stimulate cutaneous wound healing [3, 5, 59–61], achieving good and fast healing with the final presence of a scar; however, in our study, we observed that the skin regenerates with similar characteristics to the normal skin and presents a small scar and that with time the epithelium will become normal following the repair process as mentioned by Nuschke et al. [62]. However, there are clinical studies that use autologous fat grafting to correct wound scars, demonstrating that they are able to stimulate the regeneration process [63–65].

The therapeutic potential of autologous MSCs has been demonstrated, both in preclinical and clinical studies, in different pathologies. However, in many occasions, the use of autologous transplantation can have some limitations regarding to the MSC production, the method for therapy, the delivered dose, the stage of the disease, and the status and/or genetic receptivity of the patient [66, 67].

On the other hand, recent clinical studies have demonstrated therapeutic efficacy in androgenic alopecia, obtaining greater hair density by transplanting autologous ASC, PRP, stromal vascular fraction (SVF), and micrografts containing mesenchymal stem cells from the hair follicle [68–71] and also by topical use [72]. These interesting results would allow the possibility of using our methodology in pathologies in which the hair follicle is damaged or absent.

As outlined above, the consideration of using autologous or allogenic mesenchymal cells is still under discussion. This approach must be done in terms of the clinical benefit that will be obtained. From our point of view, allogeneic mesenchymal cell therapy is of great value in the tissue regeneration process.

In this study, we analyzed the gene expression of *IL10*, *MMP-2*, *GM-CSFF*, and *VEGFA* in the process of cutaneous wound healing in a canine model treated with allogeneic ASCs. These gene products have important biological effects on wound healing [23, 73].

It is well known that the process of wound healing is an organized event resulting in the restoration of the skin. It involves the interactions of many different cell types, matrix components, and biochemical factors. In this context, the *GM-CSF* gene is a possible candidate for the regulation of wound healing because it is synthesized by a number of cells involved in the repair process [74–76].

Consistent with these comments, we found an upregulation of *GM-CSF* at the gene level in our canine model treated with ASCs. GM-CSF has been shown to exert beneficial effects on wound healing in patients suffering from poorly healing wounds and chronic skin ulcers with diverse etiology [23, 77].

Considering the role of GM-CSF in transmigration (Fig. 2), the increased gene expression levels of *GM-CSF* may contribute to the recruitment of cells that participate in skin repair to the site of injury.

However, for *IL-10* and *MMP-2* genes, there were no significant differences between treatments and between them; a possible explanation is that IL-10 regulates MMP-2 expression [73, 78].

With respect to *VEGFA* gene, we expected an increase in gene expression, taking into consideration the histological findings which revealed better organization of re-epithelialization; on the contrary, a significant decrease was found at day 10 post-initial wounding. Ebrahimian et al. demonstrated that ASC treatment promotes angiogenesis and accelerates wound healing by producing VEGF protein [79]. He showed an increase in VEGF on day 7 after wound initiation, while on day 10, the VEGF value decreased, which is similar to our results. On the other hand, Kanji et al. hypothesize that MSCs would behave like pericytes, which stabilize blood vessel formation [80].

In view of the data we present in this study, we suggest that the beneficial effects observed in canine wounds after allogenic ASC therapy are due not only to direct ASC action but also to indirect paracrine processes through the induction of secondary factors involved in wound repair.

## Conclusion

To the best of our knowledge, the study we have developed in this paper represents, for the first time, a cell therapy with allogeneic ASCs in patients with acute and chronic wounds. It is important to note the clinical safety of our protocol; an important conclusion is that this study represents a translational model for human wound research with respect to tissue regeneration. The development of an allogeneic ASC therapy to improve wound healing could have clinical impacts for both dogs and people.

## Data Availability

The datasets generated and/or analyzed during the current study are available from the corresponding author on reasonable request.

## Abbreviations

ARRIVE: Animal Research: Reporting In Vivo Experiments
ASCs: Adipose mesenchymal stem cells
AT: Adipose tissue
CM: Conditioned medium
GM-CSF: Granulocyte-macrophage colony stimulating factor
HGs: Housekeeping genes
IL-6: Interleukin-6
IL-7: Interleukin-7
IL-8: Interleukin-8
IL-10: Interleukin-10
IL-15: Interleukin-15
IL-18: Interleukin-18
IP-10: Interferon ɣ-inducible protein-10
KC: Keratinocyte chemoattractant
MCP-1: Monocyte chemoattractant protein-1
MSCs: Mesenchymal stem cells
PBS: Phosphate-buffered saline
PRP: Platelet-rich plasma
SVF: Stromal vascular fraction
TNFα: Tumor necrosis factor α
